# No Diagnostic Value of Plasma Clusterin in Alzheimer's Disease

**DOI:** 10.1371/journal.pone.0050237

**Published:** 2012-11-28

**Authors:** Edina Silajdžić, Lennart Minthon, Maria Björkqvist, Oskar Hansson

**Affiliations:** 1 Brain Disease Biomarker Unit, Department of Experimental Medical Science, Wallenberg Neuroscience Center, Lund University, Lund, Sweden; 2 Clinical Memory Research Unit, Department of Clinical Sciences Malmö, Lund University, Malmö, Sweden; 3 Memory Clinic, Skåne University Hospital, Malmö, Sweden; “Mario Negri” Institute for Pharmacological Research, Italy

## Abstract

There is an urgent need for biomarkers to enable early diagnosis of Alzheimer's disease (AD). It has recently been shown that a variant within the clusterin gene is associated with increased risk of AD and plasma levels of clusterin have been found to be associated with the risk of AD. We, therefore, investigated the diagnostic value of clusterin by quantifying clusterin using an ELISA in plasma from 171 controls, 127 patients with AD, 82 patients with other dementias and 30 patients with depression. We observed similar plasma clusterin levels in controls, AD patients and patients with other dementias, suggesting that plasma clusterin levels have no diagnostic value for AD. There was a slight, but significant, increase in plasma clusterin in patients with depression compared to all other groups tested, which may warrant further investigation.

## Introduction

As populations get older, the prevalence of Alzheimer's disease (AD), the major cause of dementia, will increase considerably during the coming decades [1]. Disease-modifying therapies for AD are more likely to be successful if initiated during the early stages of the disease when the neurodegeneration is not yet too severe [2], [3]. Biomarkers are, therefore, urgently needed to correctly identify subjects affected by AD before they have developed dementia and to track disease progression in AD [4].

Recently, several independent genome-wide association studies have identified the CLU gene, encoding clusterin, as a genetic locus associated with AD [5], [6], [7] raising hope for clusterin as a potential marker of AD. Clusterin, also known as apolipoprotein J, is a multifunctional lipoprotein involved in amyloid-β (Aβ) fibrillisation and clearance [8], [9] complement inhibition [10], [11] and neuronal apoptosis [12], [13]. Clusterin is expressed in many tissues but the expression is particularly high in the brain [14].

Several studies have examined the potential of plasma clusterin as a biomarker for AD [15], [16], [17], [18]. Thambisetty *et al* (2010) found no difference in plasma clusterin between controls and AD subjects but found clusterin levels to be associated with entorhinal cortex atrophy, baseline disease severity and rapid clinical progression in AD [17]. Schrijvers *et al* (2011) also found that clusterin levels were associated with increased disease severity, as measured by MMSE, and that plasma clusterin was higher in AD patients than healthy controls, however, increased levels of clusterin did not precede the development of AD, suggesting that clusterin is not a potential early marker of subclinical disease [15]. Thus, the association of plasma clusterin with prevalence and severity of AD [15], [16], [17] and its correlation with brain atrophy in mild cognitive impairment [18] suggests that plasma clusterin may be a potential marker of disease progression in AD.

Recent data show that highly cited biomarker studies often report larger effect estimates than are reported in subsequent meta analyses [19], highlighting the importance of further validation in future biomarker research. Therefore, in the present study, we evaluated the diagnostic value of clusterin using plasma samples obtained at Skåne University Hospital, Sweden, from 171 controls, 127 patients with AD, 30 patients with depression, and 82 patients with other types of dementia (34 with Lewy Body dementia (DLB), 12 with Parkinson's disease with dementia (PDD), 17 with frontotemporal dementia (FTD), and 19 with vascular dementia (VaD)).

## Methods

### Collection and processing of human plasma samples

The subjects in this study were recruited at the memory disorder clinic, Skåne University Hospital, Malmö, Sweden. All patients underwent thorough standard examinations conducted by a trained physician, including neurological, physical and psychiatric examinations. All patients diagnosed with AD had to meet the DSM-IIIR criteria of dementia [20] and the criteria of probable AD defined by NINCDS-ADRDA [21]. Patients diagnosed with VaD fulfilled the DSM-IIIR criteria of dementia and the requirements for probable VaD by NINDS-AIREN [22] or the recommendations by Erkinjuntti *et al.* for VaD of the subcortical type [23]. For the diagnosis of DLB or FTD, the consensus criteria by McKeith *et al.* [24] and McKhann *et al.* were used [25], respectively. The healthy volunteers had no memory complaints or other cognitive symptoms, and no active neurological or psychiatric diseases.

Non-fasting plasma was collected between 9 and 11 am. After venipuncture, blood was collected in tubes prepared with EDTA to prevent coagulation. Samples were centrifuged, and plasma was removed from the tubes leaving 1 ml of plasma to avoid contamination of plasma with blood cells. Within one hour of venipuncture the plasma was frozen in polypropylene tubes at −80°C until biochemical analysis. The study was conducted in accordance with the Helsinki Declaration and approved by the ethics committee of Lund University, Sweden. Plasma sampling, in demented cases, was carried out as part of clinical routine, and some of the plasma samples were saved in a biobank for future analyses with the subjects and/or their relatives giving informed consent for research using these samples. All individuals gave informed consent either by use of a passive consent procedure where consent for retrospective use of banked clinical samples and data was assumed if individuals did not actively retract permission, as instructed in local press advertisements, or by active written informed consent. All of our consent procedures were approved by the Lund University ethics committee.

### Analysis of plasma clusterin

Clusterin plasma levels were measured in duplicate using a Human Clusterin ELISA (BioVendor, Heidelberg, Germany) that has previously been used in numerous publications [26], [27]. Spiking and recovery experiments showed 102% recovery and inter-assay variability was less than 9%. Quality control values were within expected range.

### Statistical analysis

Inter-group differences in plasma clusterin were identified using a nonparametric Kruskal-Wallis test followed by a Mann-Whitney test on each pair of groups, adjusting the p-value using the Bonferroni method. Linear regression analyses were used to investigate associations between plasma clusterin and Mini-Mental-State Examination (MMSE) in all clinical groups, whereas correlations between plasma clusterin and cerebrospinal fluid biomarkers Aβ_42_, total tau and phosphorylated tau were only performed in 31 AD samples.

## Results

**Figure 1:**
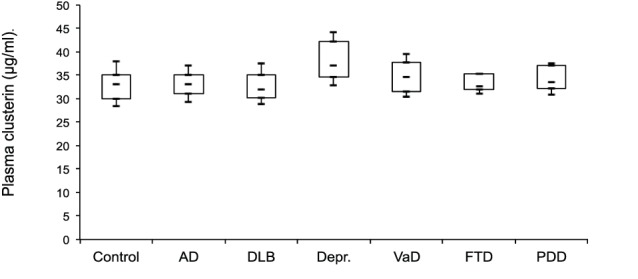
Box-and-whisker plots of plasma clusterin concentration. The boxes represent the 25% quartile, median and 75% quartile and the whiskers show the mean +/− standard deviation. Plasma clusterin was significantly higher in depressed patients compared to controls, AD, DLB, and FTD subjects (p<0.001).

In table 1 we present the demographic data and the plasma clusterin levels. Clusterin plasma levels did not differ between controls and patients with AD, DLB, VaD, FTD or PDD. A small, but significantly different increase in plasma clusterin was observed in patients with depression when compared to controls, AD, DLB, and FTD subjects (p<0.001) (see Figure 1).

**Table 1:** Subject demographics and plasma clusterin levels.

	Controls (n = 171)	AD (n = 127)	DLB (n = 34)	Depr. (n = 30)	FTD (n = 17)	VaD (n = 19)	PDD (n = 12)
**Mean age, years (range)**	74 (62–99)	75 (56–87)	75 (54–85)	59 (42–76)	61 (45–75)	76 (56–87)	72 (62–81)
**% of females**	64	71	68	50	41	68	42
**MMSE**	29±0.1	21±0.4	21±0.89	28±0.4	22±1.8	22±0.9	20±1.9
**Clusterin, µg/ml [SD]**	33.3 [4.8]	33.3 [3.9]	33.2 [4.4]	38.6 [5.6]*	33.3 [2.1]	35.0 [4.5]	34.1 [3.4]

* p<0.001 when comparing Depr. with controls, AD, DLB or FTD using Kruskal–Wallis one-way analysis of variance by ranks followed by Mann-Whitney U tests.

**Figure 2:**
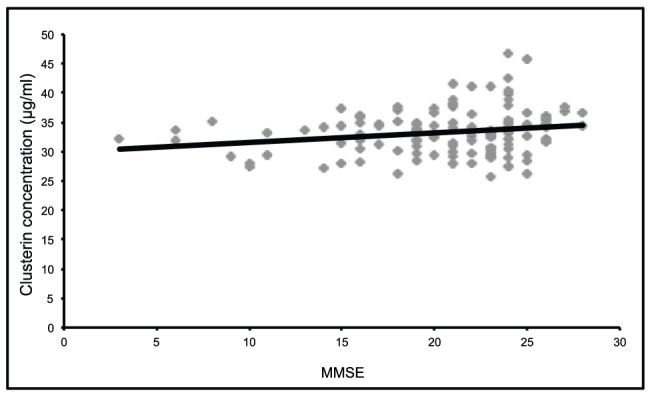
Correlation between plasma clusterin levels and Mini-Mental-State Examination (MMSE) scores in AD patients. The Pearson correlation (adjusting for age and gender) between clusterin concentration and MMSE equals 0.196 suggesting that levels of clusterin weakly correlate with improved cognition as demonstrated by increasing MMSE score (p<0.05).

We examined the correlation between plasma clusterin and MMSE in each group, individually (AD, DLB, PDD, FTD, VaD and depression). As can be seen in Figure 2, in the AD group, there was only a weakly positive correlation between plasma clusterin and MMSE score, a measure of global cognition (r^2^ = 0.041, p-value 0.03). There was no correlation between clusterin levels and MMSE in any other group tested. We also examined the correlation between plasma clusterin and CSF biomarkers Aβ_42_, total tau and phosphorylated tau in the AD group, however, we did not observe significant correlations (data not shown).

## Discussion

In recent genome-wide association studies, clusterin has been identified as a risk factor for AD [5], [6], [7]. Currently available cerebrospinal fluid markers can identify prodromal AD with acceptable accuracy [28], [29]. However, plasma is easier to obtain than cerebrospinal fluid and supported by the genomic studies, studies proposing clusterin as a potential plasma marker identifying AD [15], [16] have created a large interest.

As a result, several studies have recently investigated peripheral clusterin levels both in presymptomatic and symptomatic AD. IJsselstijn *et al.* showed that there was no significant difference in clusterin levels between presymptomatic AD subjects and controls [30]. Two recent studies have reported increased plasma clusterin levels in AD relative to healthy controls [15], [16]. Contrastingly, two other studies found no difference in clusterin levels between healthy controls and AD patients [17], [27]. In the current study, we did not observe increased plasma levels in AD subjects compared to controls and we have found that plasma clusterin levels could not discriminate AD subjects from healthy controls, nor with subjects with other dementias.

Thambisetty and coworkers showed, in 2010, that higher concentrations of plasma clusterin were associated with greater atrophy of the entorhinal cortex in AD. By contrast, in 2012, Thambisetty and coworkers found that in mild cognitive impairment, higher plasma clusterin levels were associated with slower rates of brain atrophy [18]. Our findings demonstrate no inverse correlation between clusterin levels in plasma and cognitive performance in individuals with AD, in contradiction to recent publications showing plasma clusterin to associate with both prevalence and severity of AD [15], [16], [17]. We observed a weakly positive association between plasma clusterin and MMSE, suggesting a weakly negative association between plasma clusterin and severity of cognitive impairment.

Interestingly, in the present study, plasma clusterin levels were significantly increased in subjects with depression. Although there are no previous reports linking clusterin to depression, our finding that clusterin is increased in subjects with depression ties in with the growing evidence on inflammatory markers as potential markers for depression. Several studies consistently report that subjects with depression demonstrate increased plasma levels of a variety inflammatory biomarkers when compared with nondepressed subjects [31], [32].

Interestingly, increased levels of the complement factors C3 and C4 have been observed in patients with depression compared to controls [33], [34]. It would, therefore, be interesting to investigate whether the increased clusterin levels that we observe in depressed subjects are linked to increased complement activation. Although the finding of increased plasma clusterin in depression fits in with the hypothesis of depression being an inflammatory disorder, it is necessary to replicate this finding in a different and larger cohort.

## Conclusions

Biomarkers are urgently needed to correctly identify subjects affected by AD, track disease progression or measure response to treatment and characterising protein markers in plasma has created optimism for finding easily accessible and detectable disease-specific markers. However, our results suggest that plasma clusterin levels have no diagnostic value for AD. In our study, plasma clusterin was significantly elevated in depressed subject compared to controls and several dementias, adding to the evidence of involvement of inflammation in depression.
